# Formation and Investigation of Physicochemical and Microbiological Properties of Biocomposite Films Containing Turmeric Extract Nano/Microcapsules

**DOI:** 10.3390/polym15040919

**Published:** 2023-02-12

**Authors:** Natalia Stanisławska, Gohar Khachatryan, Karen Khachatryan, Magdalena Krystyjan, Małgorzata Makarewicz, Marcel Krzan

**Affiliations:** 1 Students’ Scientific Society of Food Technologists, Faculty of Food Technology, University of Agriculture, ul. Balicka 122, 30-149 Kraków, Poland; 2 Faculty of Food Technology, University of Agriculture, ul. Balicka 122, 30-149 Kraków, Poland; 3 Jerzy Haber Institute of Catalysis and Surface Chemistry, Polish Academy of Sciences, 31-120 Kraków, Poland

**Keywords:** biocomposite, nanocapsules, curcumin, innovative packaging

## Abstract

In the era of growing plastic consumption, food waste by consumers and overproduction caused by economic reasons, the global goal is to decrease these phenomena. Biocomposite films investigated in the past years are creating a promising future toward ecological, intelligent and active packaging. Due to their unique properties, they can be used in many areas of our life and reduce the constantly increasing pollution of our planet. The aim of our study was to obtain innovative and flexible biopolymer films based on sodium alginate and chitosan, as well as to develop methods for generating nanocapsules with turmeric extract in them. Bionanocomposites were analyzed using UV-VIS, FTIR, photoluminescence spectroscopy and SEM microscopy, while contact angles, surface free energy, particle size (DLS) and zeta potential were determined. The mechanical and colorimetric properties of the produced films were investigated, and the water content, solubility and water absorption were determined. Microbiological tests were carried out to analyze the influence of the produced films on the development of microorganisms. The results of the performed analyses allowed us to confirm the presence of curcumin nano- and microcapsules in the alginate–chitosan composite. Moreover, studies have shown that the structure of polysaccharides does not change during capsule manufacturing. The film with the highest concentration of the capsules showed better parameters in tests of solubility, water content, degree of swelling and mechanical properties. The obtained properties of the developed films allow them to be used as active and intelligent packaging materials, or as their parts.

## 1. Introduction

A key element in ensuring food safety is packaging. This is designed to protect food from harmful environmental influences (moisture, temperature), biological hazards (microorganisms, pests) and mechanical damage (impact, pressure). For most food products, it is essential both to ensure the right quality and safety during storage and transport, with the aim of extending the shelf life. Packaging also provides product information, such as composition, origin, nutritional value and minimum durability date.

With their good protective properties, plastics dominate the packaging market, being cheap and so far irreplaceable, although they have negative environmental impacts. This is why alternatives are increasingly being sought to produce packaging using substances of natural origin that will reduce harmful effects on the ecosystem [1,2,3,4]. All these factors are driving the development of innovative packaging to maintain and monitor food safety, extend the shelf life and reduce the production of non-biodegradable waste [5]. These are the characteristics of smart packaging, i.e., active and intelligent packaging. Polymer nanocomposites, obtained by dispersing modifiers with dimensions of a few to several hundred nanometres in a polymer matrix, show great potential in this field. Even a small amount of nanoadditives is able to significantly improve the mechanical, optical, electrical, thermal and bacteriostatic properties of a composite material [6,7,8,9]. Selecting the right ingredients for nano/microcomposites and improving their manufacturing methods are important parts of enabling technological development [3].

When it comes to the production of innovative packaging, biopolymer films, which are a mixture of polysaccharides, are gaining popularity. Due to their easy availability and low production costs, they have great potential for applications in many industrial sectors. Furthermore, the diversity of functional groups makes them particularly amenable to modification [9,10,11]. Among the polysaccharides used for manufacturing composites, chitosan and alginate are attracting much attention. In addition to a number of benefits characteristic of all sugar polymers, such as biodegradability, bioavailability and membrane-forming ability, they exhibit a number of unique physicochemical and functional properties [12,13,14,15,16,17,18,19]. They form a good barrier, preventing the loss of volatile flavor compounds and providing protection against microorganisms and viruses.

The problem is that their tensile strength, structural strength and physical properties, such as water-vapor permeability after film manufacture, do not fully meet the requirements for food packaging. Promising effects are achieved by the formation of nanocomposites and their combination with biological extracts in the form of nano/microcapsules.

Curcumin (1,7-bis(4-hydroxy-3-methoxyphenyl)-1,6-heptadiene-3,5-dione) is the main polyphenol in the rhizome of Curcuma longa, known as turmeric. Turmeric is known worldwide, primarily for its use as a spice included in curry mixes and as a yellow coloring agent. It has also equally valuable, if less popular, preservative, antimicrobial, antioxidant and even medicinal properties. The medicinal effects of turmeric have not yet been confirmed by clinical studies, but the properties of its main active ingredient have been of interest to scientists for many years [20,21,22]. Current research, conducted by many scientists, seeks to determine the scope of curcumin’s action in the prevention and treatment of diseases, with a view to developing effective and non-toxic drugs that could replace modern pharmaceuticals and minimize unwanted side effects of therapy. Curcumin has been proven to have antioxidant, antiviral and anti-inflammatory effects [23,24]. Considering the use of curcumin as an ingredient in active films, its antimicrobial properties have attracted the most attention. Tosati et al. have developed edible films containing curcumin extract, showing that their use in sausages effectively prevents the growth of *Listeria innocua* [25]. The antibacterial and preservative properties of curcumin offer a whole range of possibilities for its use as an ingredient in active food packaging [26]. Significantly, since it changes color under the influence of pH, it may also become an indicator of product freshness. Its acidic pH gives it a light yellow color, while its color in an alkaline environment is strong red. This property makes it a good indicator of spoilage in, for example, shrimps, which produce volatile amines during decomposition, so that the pH changes to alkaline and the color of the film becomes red [27].

The aim of the present study was to obtain films based on chitosan and sodium alginate, containing curcumin nanocapsules, and to investigate their physicochemical and antimicrobial properties in order to assess their suitability for application in active packaging. The study investigated the antimicrobial and antifungal activity with respect to selected strains of microorganisms and the biodegradation rate of the composites produced in soil.

## 2. Materials and Methods

### 2.1. Materials

The following chemical reagents were used to produce the nanocomposites: chitosan (high molecular weight: 310,000–375,000 Da, degree of deacetylation >75%) from shrimp shells, (Sigma-Aldrich, Saint Louis, MI, USA), sodium alginate (Sigma-Aldrich), acetic acid (Chempur, 99.5%), glycerine (99.5%, Chempur), deionized water, extra virgin olive oil, turmeric, ethanol 96% p.a. grade.

The following microbial strains were used for microbiological testing: *Escherichia coli* (DSMZ 1116), *Aspergillus fumigatus* (LOCK 042600), *Penicillium expansum* (LOCK 0535). The growth mediums used in the experiment: Mueller Hinton Lab-Agar (BIOMAXIMA), DRBC Lab-Agar (BIOMAXIMA).

### 2.2. Films Preparation

#### 2.2.1. Method for Obtaining Turmeric Extract

The ground turmeric rhizome was extracted with ethanol using a Soxhlet extractor. The extraction lasted approximately 5 h. A total of 1 g of the extract contains 4 mg of curcumin.

#### 2.2.2. Preparation of Emulsion with Curcumin Nanocapsules

A total of 32 g of emulsion containing curcumin nanocapsules was prepared by placing 8 g of demineralized water, 8 g of curcumin extract and 16 g of olive oil in a conical flask (50 mL). This flask was then placed in an ultrasonic bath cooled in an ice bath (at a temperature of 2 °C). The mixture was exposed to ultrasound, at 40 kHz, for 25 min.

#### 2.2.3. Preparation of the Chitosan–Alginate Matrix

A suspension of 56 g of sodium alginate in 2716 g of water was prepared. The resulting suspension was stirred on a magnetic stirrer at 70 °C, 700 rpm, until a homogeneous gel was obtained. Then 28 g of glycerine was added as a plasticizer. A 2% solution of sodium alginate was thus obtained.

Similarly, 1400 g of 1.5% chitosan solution was prepared by weighing out 21 g of chitosan and dissolving it in 1368.5 g of 2% acetic acid. The mixture was placed on a magnetic stirrer (700 rpm, temperature: 70 °C), and then 10.5 g of glycerine was added as a plasticiser.

The polymers gels were combined at a 2:1 weight ratio of alginate: chitosan, using a homogenizer (Polytron PT 2500 E, Kinematica AG, Malters, Switzerland). The gel was treated as a polymer matrix in subsequent steps. In order to select the best concentration of the active ingredient, three samples with different concentrations of curcumin were prepared and further analyzed.

#### 2.2.4. Preparation of the Films

The previously prepared matrix was mixed with demineralized water and emulsion containing curcumin nanocapsules (Table 1), with a homogenizer. The obtained gel was poured into 240 × 350 mm rectangular stainless-steel trays, 200 g each. All the films were dried for two days at room temperature (24 °C) and a humidity of approximately 45%. After drying, the films were detached from the trays and placed in tightly sealed string bags until analysis.

### 2.3. Films Characterization

#### 2.3.1. FTIR Spectroscopy

The FTIR spectra of the fabricated composites were analyzed in a wavelength range of 4000–700 cm^−1^ using a MATTSON 3000 FTIR spectrophotometer (Madison, WI, USA), equipped with a 30SPEC 30 Degree Reflectance accessory (MIRacle ATR, PIKE Technologies Inc., Madison, WI, USA).

#### 2.3.2. UV-VIS Spectroscopy

The UV-Vis absorption spectra of all the prepared films were analyzed using a Shimadzu 2101 scanning spectrophotometer (Shimadzu, Kyoto, Japan) in the wavelength range 200–700 nm.

#### 2.3.3. Photoluminescence Spectroscopy

Photoluminescence measurements for the films were carried out at room temperature using a HITACHI F7000 spectrophotometer (Hitachi Co. Ltd., Tokyo, Japan). The emission spectra of the film were measured using an excitation wavelength of 360 nm.

#### 2.3.4. Determination of Water Content, Solubility and Degree of Swelling

Squares of 2 × 2 cm were cut from the control, Cur-1, Cur-2 and Cur-3 samples and weighed on an analytical balance (m_1_). The samples were then dried in an oven at 70 °C for 24 h and weighed again (m_2_). The water content was calculated using the following formulae:

(1) $$Water\;content\;\left[\%\right]=\frac{\left(m_{1}-m_{2}\right)}{m_{1}}\;\cdot 100\%$$

Then, the squares were placed in beakers containing 30 mL of deionized water, covered and stored for 24 h at room temperature (22 ± 2 °C). The remaining water was removed and the samples were dried on the surface with filter paper and then weighed (m_3_). The remaining samples were dried in an oven at 70 °C for 24 h and then weighed (m_4_). Three measurements were taken for each sample and the average value of the parameter was determined. These solubility and degree of swelling were calculated using the following equation [28]:

(2) $$Solubility\;\left[\%\right]=\frac{\left(m_{2}-m_{4}\right)}{m_{2}}\;\cdot 100\%$$

(3) $$Degree\;of\;swelling\;\left[\%\right]=\frac{\left(m_{3}-m_{4}\right)}{m_{3}}\;\cdot 100\%$$

#### 2.3.5. Mechanical Tests

The analysis was performed in accordance with ISO Standards [ISO 527-1:2019]. The films were cut into 35 × 6 mm strips and placed in grips. The initial distance between the grips was 20 mm and the peel rate was 2 mm/min. Tensile strength (TS) was calculated by dividing the maximum force at break of the film by the cross-sectional area of the film. The percent elongation at the break (EAB) was calculated by dividing the elongation at the break point by the initial measurement length and multiplying by 100. The results reported were the averages of ten repetitions.

#### 2.3.6. Film Color Measurement

The color of the film surface was measured with a Konica MINOLTA CM-3500d (Konica Minolta Inc., Tokyo, Japan), using the standard illuminant D65/10° observer with a 3 mm diameter window. The results were expressed using the CIELab system. The following parameters were determined: L* (L* = 0 black, L* = 100 white), a*—the proportion of green (a* < 0) or red (a* > 0), b*—the proportion of blue (b* < 0) or yellow (b* > 0) [10]. The measurements were taken on a white standard background. The experiment was repeated 5 times.

#### 2.3.7. Opacity of Films

The degree of UV impermeability of the films was measured by exposing the film sample to absorption of 600 nm light from a Helios-Gamma 100–240 UV/V spectrophotometer [29]. Rectangular film samples were placed directly into the test cell of the spectrophotometer. The empty test cell served as a reference. The opacity (O) of the films was calculated according to the equation:

O = A_600_/x, (4)

where A_600_ is the absorbance at 600 nm and x is the film thickness [mm]. A higher O value indicated a higher degree of opacity/opacification of the sample. The analyses were performed in five replicates.

Based on the above analyses, the film with the best properties (Cur-2) was selected and further tests were performed on the control film and the film with the selected concentration.

#### 2.3.8. Scanning Electron Microscopy

The size and morphology of the prepared nanoparticles were analyzed using a JEOL JSM-7500F high-resolution scanning electron microscope (SEM) (Akishima, Tokyo, Japan).

#### 2.3.9. Determination of the Wetting Angles

The wetting angles were determined using a Kruss-DSA100M device (Kruss GmbH, Hamburg, Germany). The contact angles of distilled water and pure diiodomethane on the tested surfaces of the polysaccharide films were determined using the stalagmometric method. All the measurements were taken in an environmental chamber, under constant temperature (22 ± 0.3 °C) and constant humidity conditions (40 ± 5%). The wetting angles were measured using a device that allowed photographs of the deposited droplet to be taken to a suitable approximation, and then the angles formed by the flat surface of the film and the plane tangent to the surface of the liquid bordering it were determined using DSA4 computer software (Kruss GmbH, Hamburg, Germany).

#### 2.3.10. Surface Free Energy (SFE)

Surface free energy was investigated using the Owens–Wendt method [30]. Two liquids, bipolar water and polar diiodomethane, were used to characterize the surface free energy of the examined hydrogels. The method assumes that the interactions between the molecules of two substances present in their surface layer are equal to the geometric average of the intermolecular interactions within each substance. A detailed introduction to this method was presented by Rudawska et al. [31].

#### 2.3.11. Particle/Aggregate Size (DLS) and Zeta Potential

The zeta potential and particle/aggregate sizes were measured using a Malvern Zetasizer Nano ZS with disposable measurement cuvettes (DTS 1065, Malvern). The zeta potential was calculated from the electrophoretic mobility of the particles using the Smoluchowski model. The results were expressed as the average of measurements in 20 consecutive series. All the measurements were taken in aqueous mixtures obtained after dissolving the unwrapped packaging film in 1 wt. % acetic acid (this method was used to completely dissolve the chitosan). Film samples of 0.1 g (± 0.01 g) were dissolved in 5 mL of 1% wt. aqueous acetic acid solution. A magnetic stirrer stirred the mixtures for 1 h until the films were completely dissolved.

#### 2.3.12. Antimicrobial Activity of the Films (Disc Diffusion Test)

**Figure 1:**
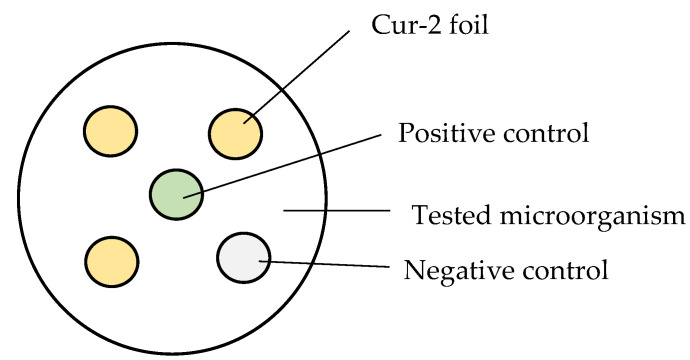
Scheme of the placement of the film discs on nutrients.

The antimicrobial activity of the alginate–chitosan film with curcumin was tested against the bacteria *Escherichia coli* (DSMZ 1116) and the fungi *Penicillium expansum* (LOCK 0535) and *Aspergillus fumigatus* (LOCK 04260), which are common causes of the microbial contamination of food products. For the determination of antimicrobial activity, the spread plate technique was employed for pure bacterial cultures (OD 8.3 × 10^8^ jtk/cm^3^) or plate cultures of filamentous fungi with a sterile swab onto Mueller Hinton Lab-Agar medium (BIOMAXIMA)—*E. coli* or DRBC Lab-Agar medium (BIOMAXIMA)—molds. Next, discs cut from the test films with a diameter of 1 cm were placed on the surface of the inoculated culture medium (Figure 1). The negative control was the film without curcumin, while the positive control was commercial tissue paper discs soaked in Penicillin G 10U (BIOMAXIMA) (bacteria) or Nystatin 100U (BIOMAXIMA) (molds). After the incubation time (24–48 h, 37 °C (bacteria), 72–96 h, 25 °C (fungi)), the diameter of the zones of inhibition (mm) was measured. All analyses were performed in triplicate.

#### 2.3.13. Evaluation of the Susceptibility of the Tested Films to Soil Microorganisms Degradation

Preliminary tests aimed at determining the susceptibility of the tested films to degradation with the participation of soil microorganisms were performed by cutting 1 × 4 cm strips of the control film and the film with the active substance, and then placing them in glass dishes filled with garden soil so that 1 cm of the film in question protruded above the soil surface.

Prepared in this way, the samples were placed in a dark plastic bag together with a beaker of distilled water to preserve the appropriate humidity during the process. The tightly closed bag was stored at room temperature. All analyses were performed in triplicate. The degree of structure damage of the film was assessed after 30 and 60 days of storing the samples. Macroscopic changes (etchings, color changes, etc.) were determined using a MSZ 200 stereo microscope.

#### 2.3.14. Statistical Analysis

Experimental data were analyzed in terms of variance, with a confidence level of *p* = 0.05, using a Statistica v. 8.0 (Statsoft, Inc., Tulsa, OK, USA). The Fisher test was used to determine statistically the significant differences.

## 3. Results and Discussion

Measurements of the UV-Vis, ATR-FTIR and emission spectra were performed to compare the degree of encapsulation.

**Figure 2:**
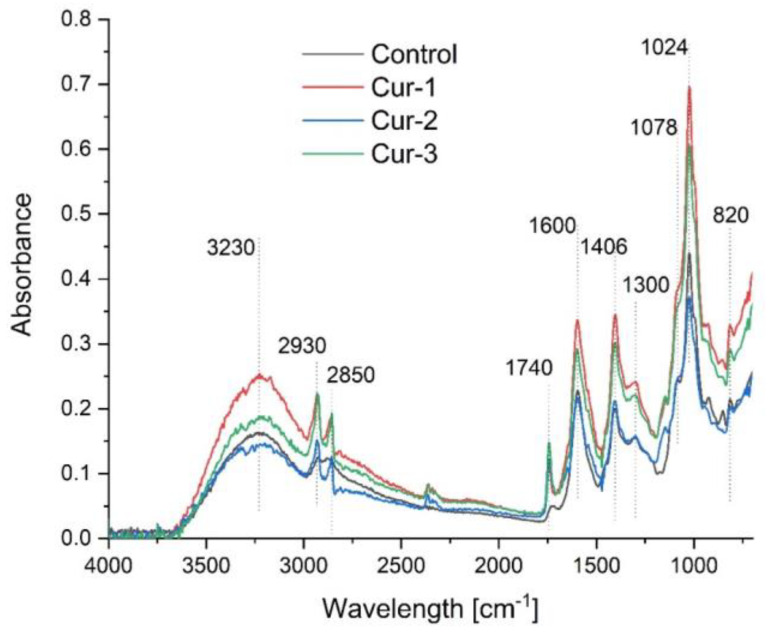
ATR-FTIR spectra of the control film and composites containing nanocapsules.

In order to determine possible chemical changes and interactions between the components in the obtained films, ATR-FTIR spectra were performed, as shown in Figure 2. The control film consists mainly of chitosan and sodium alginate. The other composites contained the addition of a nanoemulsion containing curcumin and olive oil. The ATR-FTIR spectra of the resulting composites contained characteristic bands for chitosan: bands at 3000 to 3500 cm^−1^ represent the stretching vibrations of the free hydroxyl groups and the symmetric and asymmetric N-H stretching bonds in the amine group; bands at 1078 and 1024 cm^−1^ are associated with the stretching vibrations of the -OH, 3′-OH and 5′-OH groups; extended bands at 1600 cm^−1^ (from 1550 to 1640 cm^−1^ ) are related to the stretching vibrations of the C=O group and deformation vibrations of the -NH group_2_; bands at 1406 cm^−1^ correspond to the symmetric deformation vibrations of the CH_2_ group; bands at 1022 cm^−1^ and 1300 cm^−1^ correspond to the C-O bonds and the amide group [32]. The following bands characteristic of sodium alginate were also observed in the spectra: 3230 cm^−1^ of the OH group adjacent to the amide group; and 1600 cm^−1^ and 1406 cm^−1^ asymmetric and symmetric vibrations of the COO- group, respectively. Characteristic saccharide bands were also present: at wavelengths of 1300, 1092 and 820 cm^−1^, they correspond to the C-O stretching bond vibrations, and the peak at 1032 cm^−1^ shows the CO-C bonds [33]. The characteristic peaks at about 3400 cm^−1^ (phenolic O-H stretching vibrations) were observed in composites containing curcumin, 2930 and 2850 cm^−1^ (methyl (-CH_3_) and methylene (-CH_2_) symmetric and asymmetric vibration), 1628 cm^−1^ (C=C stretching vibration in aromatic molecules), 1597 cm^−1^ (benzene ring stretching vibrations), 1740 cm^−1^ (C=O vibrations), 1510 cm^−1^ (C=C vibrations in the aromatic system), 1428 cm^−1^ (C-H deformation vibrations), 1278 cm^−1^ (aromatic C-O stretching vibrations), 1024 cm^−1^ (C-O-C stretching vibrations) [34,35,36,37].

The observed changes in the spectra of the obtained nanocomposites are due to the superposition of oil-derived bands. Strong absorption bands in the 3000–2800 cm^−1^ range are caused by the corresponding oil-derived C-H stretching vibrations. Stretching vibrations of the methylene (–CH_2_–) and methyl (–CH_3_) groups can be observed at the frequencies of 2930 and 2850 cm^−1^, respectively. The large peak around 1740 cm^−1^ is due to the stretching vibration of the C=O double bond of the oil, which overlaps with the peak from curcumin [38,39,40].

The FTIR spectra of the films with added curcumin nanocapsules do not differ in shape from those of the control film. This suggests that, as expected, the biopolymers are a carrier for the curcumin nanocapsules.

**Figure 3:**
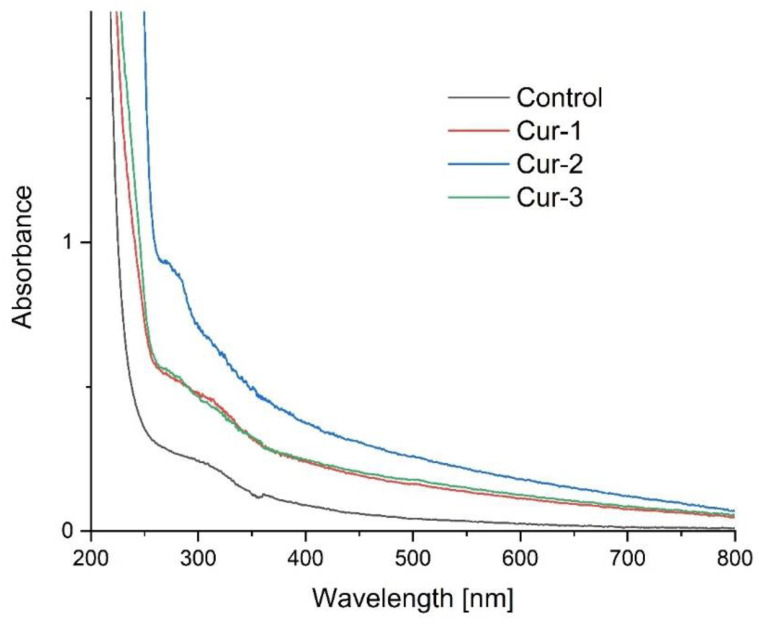
UV-Vis spectra of the control film and composites containing nanocapsules.

Figure 3 shows the absorption spectra in the ultraviolet and visible range of the resulting films. With the addition of curcumin, absorbance increases over the entire range tested, but particularly in the range from 260 to 380 nm. The Cur-2 film has the highest absorbance. Pure curcumin shows characteristic absorption bands at 250 nm and 427 nm due to π-π* and n-π* transitions. By contrast, encapsulation causes a strong absorption band to appear at about 275 nm. These results are consistent with those obtained by Omrani et al. [41], who encapsulated curcumin in cyclodextrins. They suggest that the Cur-2 film has the highest concentration of capsules, so the ratio of nanoemulsion to polysaccharides is the most optimal in this composite.

**Figure 4:**
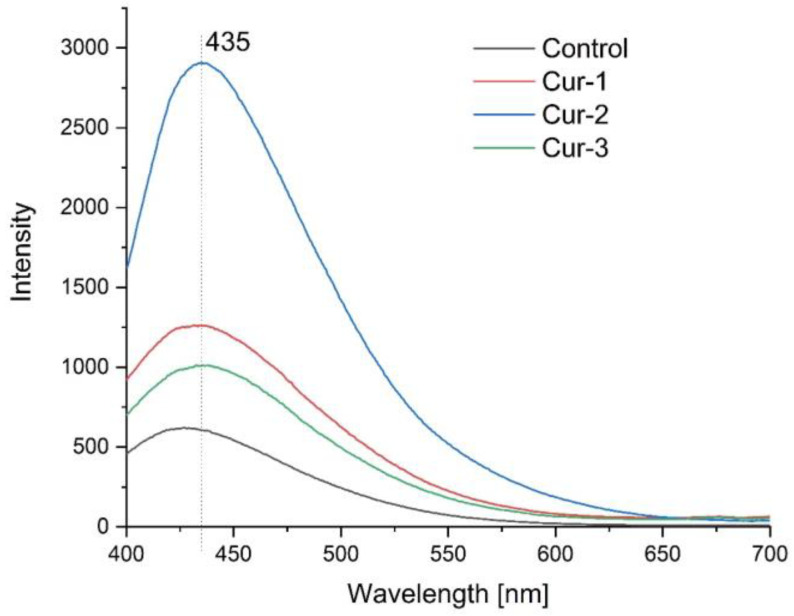
Emission spectrum of the resulting composites.

Figure 4 shows the emission spectra of the resulting films when excited with a wavelength of 360 nm. One can see a characteristic emission band for the turmeric nanocapsules at 435 nm. Numerous studies have shown that encapsulating turmeric increases the emission intensity. Bechnak et al. encapsulated turmeric in polyethylene [42], showing that the relative fluorescence yield increased by a factor of 6 in the nanocapsules.

Rahimzadeh et al. [43] synthesized curcumin nanocapsules and also found an increase in emission intensity. The relationship between emission intensity and concentration is linear only at very low concentrations, while with particle aggregation excitation radiation is unable to excite all the molecules present. The photoluminescence results obtained correlate with the UV-VIS results, indicating that the optimum concentration for curcumin encapsulation occurs in the Cur-2 composite, which has the highest emission intensity.

Table 2 shows the results of the measurements of the water content, solubility and degree of swelling of the control film and the film with curcumin added at different concentrations. The control film has the highest water content, while the Cur-2 has the lowest. The water content in the control film reaches 17%, decreasing slightly for the Cur-1 and Cur-3 film (15% and 12%). For the Cur-2 film, it is less than 10%. The solubility of the films for all samples with the addition of curcumin is not statistically different and is around 13%. This parameter is significantly higher for the control film (24.04%). This means that the addition of the active substance has a positive effect on reducing the solubility of the film. The control film had the highest value for the degree of swelling, around 31%. The samples with the addition of curcumin nanocapsules had a much lower degree of swelling: 18.63% for the Cur-1 film, while the Cur-3 and Cur-2 films were not statistically different at 12.57% and 11.22%, respectively. The best parameters for use as food packaging were the lowest possible water content, solubility and degree of swelling. Among the films tested, the Cur-2 film has the most promising properties.

Table 3 shows the mechanical properties of the film at 25 °C and 25% humidity for the control film and the film with curcumin added at different concentrations. The parameters studied were thickness, tensile strength and percentage elongation at the break point. The results illustrate that the thickness of the film increases as the concentration of the added active substance increases. For the control film, it is 0.09 mm, and for the Cur-3 film it is almost twice as much. The film thickness may enhance the opacity of samples [44], due to the increase in the solid part in the sample [10].

Tearing strength (TS) is the force required to break a section of film of specified dimensions, given in Pascals. The higher the tearing strength, the better the packaging material of the tested sample. In the case of the composites tested, the addition of curcumin adversely affects this parameter, the result for the Cur-1 and Cur-3 films being significantly lower than for the control film. Concentration 2 (Cur-2 film), however, does not cause such a large decrease; the result achieved is statistically equal to the strength of the control film. The percent elongation at the break (EAB) determines the extensibility of a given material, indicating by what percentage it can be stretched before it breaks. The elasticity of a given material is not a key property for determining whether it will perform well as packaging; still, in combination with a specific tearing strength, it provides a good basis for characterizing a given composite. For the samples tested, the elongation at break for the control film is 34.5%. The addition of curcumin at concentrations of 1 and 2 (Cur-1 and Cur-2 films) causes this value to drop to around 29%. However, it can be observed that an increase in this parameter is observed for the Cur-3 film compared to the control film (approximately 40%). From the perspective of the application of the material in packaging, the most important of the parameters studied is its high tearing strength combined with low thickness. The control and Cur-2 films have such parameters.

**Figure 5:**
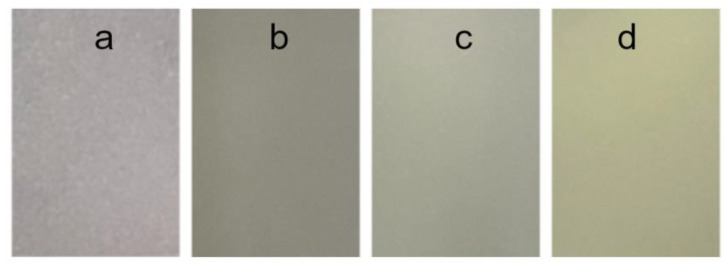
Biocomposite films: (**a**)—Control film (Control), (**b**)—Cur-1, (**c**)—Cur-2, (**d**)—Cur-3.

Table 4 shows the results of the color measurement of the individual films in the L*a*b* color space and the transparency measurement. The color of a material is defined by three components. The L* component describes the brightness (luminance) of the color from 0 to 100, where 100 is the brightest color. This component for the materials tested varies between 94.14 and 97.16. This means that all the films tested are relatively bright. The component a* represents the proportion of green or red in the analyzed color, with green shades having a negative value and red shades having a positive value. The component a* represents the proportion of blue or yellow in the analyzed color, with blue shades having a negative value and yellow shades having a positive value. The scales of the a* and b* parameters range between -150 and +100, and -100 and +150. The greatest differences can be seen in the values of the b* component. Curcumin is a yellow pigment, so the positive value of this parameter increases for films with added nanocapsules. Comparing the images of the films (Figure 5), we can observe that the last two samples (Cur-2 and Cur-3) with a higher amount of encapsulated turmeric extract were more yellow. These samples also had different values for the a* component. Statistically significant differences were observed between the films.

Transparency defines the degree of light transmission through the material without scattering. The addition of curcumin in all concentrations reduces the transparency of the film. The sample with the highest opacity was Cur-1, which is also confirmed by the film images (Figure 5). For food packaging, light-sensitive foodstuffs require less transparency, so this phenomenon may be desirable. However, it is also important to make the product visible through the packaging, so the evaluation of the film’s properties depends on its intended use [10]. The increase in the opacity of films with the addition of emulsions containing nanoparticles can be caused by the increase in film thickness, which is due to the increase in the carrier concentration [10,45,46,47].

The best parameters (degree of encapsulation, mechanical and optical properties) were displayed by the Cur-2 film, which was selected for further study.

**Figure 6:**
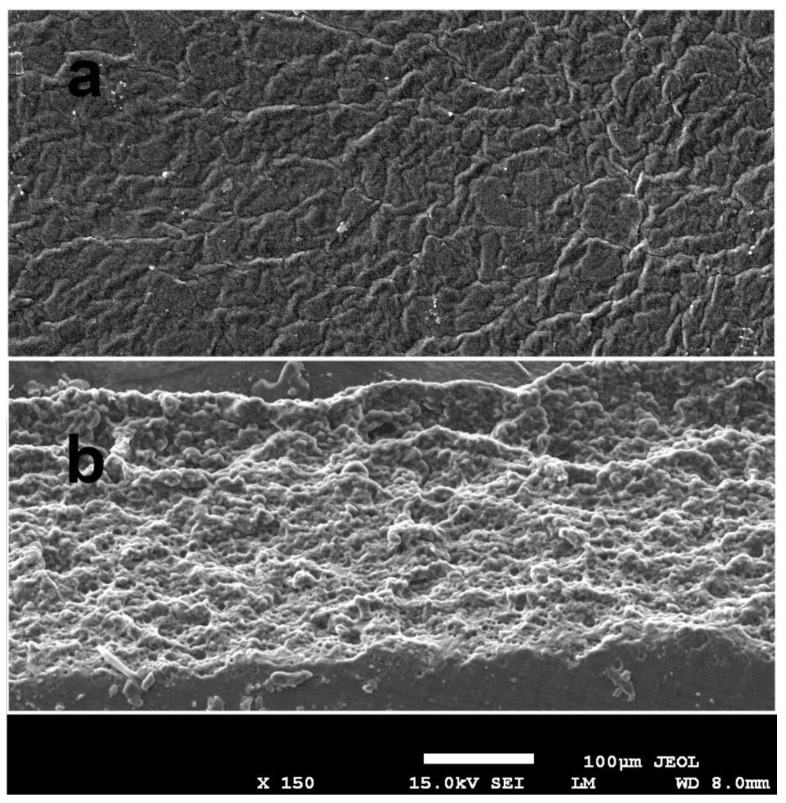
A SEM image of the Cur-2 composite on the surface (**a**) and in cross-section (**b**).

Figure 6 displays the SEM images of the finished Cur-2 film’s surface (Figure 6a) and cross-section (Figure 6b). A generally uniform surface with multiple undulations and no discernible fissures is observed. Cross-sectional imagery of the interior structure (Figure 6b) reveals a spongy one.

**Figure 7:**
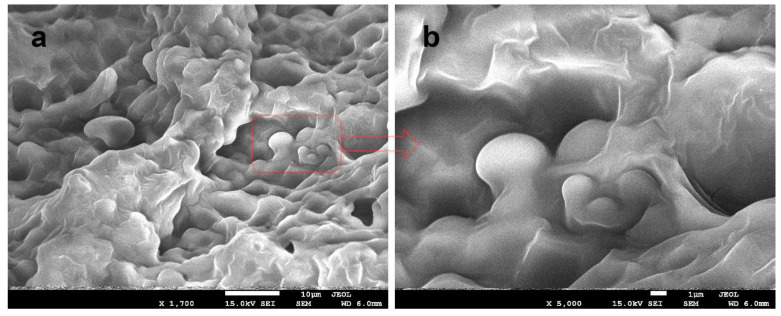
A SEM image of the Cur-2 composite at the magnifications of ×1700 (**a**) and ×5000 (**b**).

**Figure 8:**
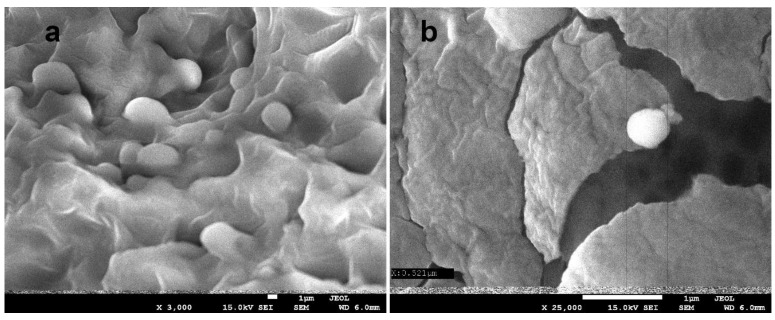
A SEM image of the Cur-2 composite at an ×3000 (**a**) and ×25,000 (**b**) magnification.

Figure 7 shows the microscopic images of the Cur-2 film taken at magnifications of ×1700 (Figure 7a) and ×5000 (Figure 7b). The images show the presence of capsules evenly distributed in the polysaccharide matrix and the presence of micropores in the internal structure. Another photograph (Figure 8) shows a microscopic image of the film in another area at the magnifications of ×3000 (Figure 8a) and 25,000× (Figure 8b). At a higher magnification (Figure 8b), the polysaccharide layer was broken and a single spherical capsule of 521 nm was visible.

The surface free energy was determined by the Owens–Wendt method [28,31]. The surface free energy of solids is an important thermodynamic parameter, making it possible to infer the adsorption of different substances on the surface of a solid and at the solid–liquid interface. The surface free energy of a solid also determines the level of adhesion of a liquid to its surface. The results of the measurements are shown in Table 5. The wetting angles for a polar liquid, such as water, indicate the hydrophilicity or hydrophobicity of the surface. The results show that the addition of the nanoemulsion with curcumin increases the hydrophilicity of the film. The wetting angle for the control sample is close to 80°, while for the sample with the active substance it decreases to 68°. The measurement for a dispersion liquid such as diiodomethane (DIM) indicated a much smaller wetting angle, 53° and 41°, respectively. The surface free energy determined based on these angles is the sum of two components: polar and dispersion. The addition of nanocapsules with curcumin results in an increase in both components of the surface free energy. It can be therefore assumed that the Cur-2 film will be better suited for bonding or printing on it, a factor that can significantly facilitate its use in packaging.

The zeta potential (ζ), or electrokinetic potential, is measured to determine the stability of the resulting nanoparticles. With an increase in the absolute value of the zeta potential, generally colloidal particles show good dispersion properties, at the same time as the electrostatic repulsion forces increase. When the zeta potential is close to zero, they become so unstable that they are prone to form aggregates. It is assumed that a result of more than +/−30 mV means that the test sample can be considered stable. The results of the measurements are shown in Table 6. The zeta potential values for the tested materials are −41.3 mV for the control film and −37.8 mV for the Cur-2 film, respectively. For both samples, the zeta potential is high enough to conclude that they are not prone to form aggregates. The addition of curcumin nanocapsules causes a slight decrease in this value. The table also includes the result of the particle size measurement using the DLS method. The particle size determined by scanning electron microscopy is about 500 nm, while the average particle size determined by DLS is much larger. It should be notated that the DLS result provides the size of the combination of nanostructures in the polysaccharide environment and, in general, the measurement results are much larger than the corresponding results obtained from electron microscopy methods.

Based on the measurements of the size of the zones of inhibition (Table 7), only the film containing curcumin nanocapsules (Cur-2) showed antibacterial properties. The zones of inhibition obtained for *E. coli* were even greater than for the positive control (penicillin G disc) (Table 7). This confirms the antimicrobial properties of curcuminoids.

Curcumin nanocapsules, slowly released from the polymer-lipid envelope, could easily penetrate the thin murein barrier in the *E.coli* cell wall and effectively inhibit its growth [48].

Varying activity results of the tested films were obtained for the tested filamentous fungi (*P. expansum*, *A. fumigatus*). Effective growth inhibition was obtained solely for *Aspergillus fumigatus,* the measured zone being comparable to the one induced by the antibiotic (positive control). The effect was significantly weaker in relation to *Penicillium expansum* (Table 7).

The antimicrobial properties of curcumin are related to its ability to disrupt the cell wall integrity of the microorganisms, synthesize reactive oxygen species and induce cellular apoptosis [49,50].

**Figure 9:**
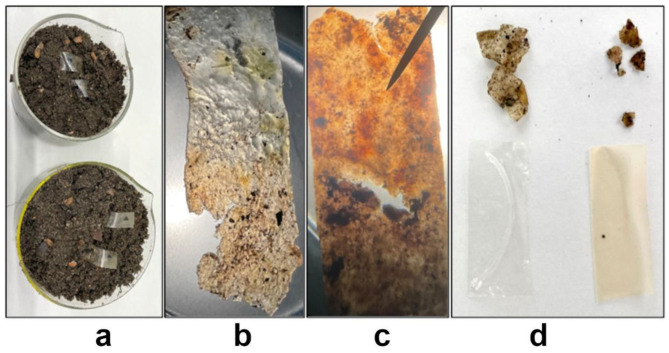
Biodegradability test of the film in soil ((**a**)—setting up the experiment, (**b**)—the control film after 30 days, (**c**)—the Cur-2 film after 30 days, (**d**)—on the left, the control film after 60 days, on the right, the Cur-2 film after 60 days).

Figure 9 shows the biodegradation effects of the tested films. After cleaning the film surface from the soil, the morphological changes in the form of cavities and intense surface discoloration were observed initially (after 30 days) on both the control film (Figure 9b) and the Cur-2 film (Figure 9c). After 60 days of storage in soil, the Cur-2 film had decomposed practically completely (Figure 9d).

In most cases, attempts to develop new biocomposites do not include degradation tests being carried out. It is difficult to perform reproducible tests of polymer biodegradation under laboratory conditions since the natural environment usually contains complex mixtures of enzymes and microorganisms that are not necessarily conducive to the degradation of a particular polymer tested, and the composition of the soil depends not only on its place of origin but also on the current weather conditions or season. All these factors vary according to the environment. Further, more detailed studies, taking into account different environments, are needed for a more precise determination of the time required for the decomposition of the prepared bionanocomposites.

## 4. Conclusions

Bionanocomposites with a smooth surface and porous structure consisting of a chitosan–alginate matrix and nanocapsules containing curcumin of approximately 500 nm were successfully produced. The analysis of FTIR, absorption and emission spectra indicate that the chemical structure of polysaccharides does not change during the production of capsules, there are no strong interactions between the curcumin nanoemulsion and the polysaccharide matrix, and the concentration of the nanoemulsion in the Cur-2 film is the most optimal. The aforementioned sample also showed better parameters in tests of solubility, water content, degree of swelling and tearing strength combined with low thickness.

The film produced has an inhibitory effect on the bacteria *Escherichia coli*, on the mold *Aspergillus fumigatus* and, to a lesser extent, on *Penicillium expansum*. These microorganisms are often present in food, so the film can serve well as active packaging.

In the soil, the tested films entirely disintegrated after 60 days.

The research carried out confirms that the presence of curcumin nanocapsules improves the properties of the chitosan–alginate film, allowing its use in packaging. Microbiological experiments prove its antimicrobial activity.

These features demonstrate the enormous potential of using nanoparticles with active ingredients in innovative packaging solutions. The nanocomposite created can be used as an active packaging material, prolonging the freshness of products.

## Figures and Tables

**Table 1:** Composition of the films.

Films	Ingredients		
	Chitosan–Alginate Matrix (g)	Demineralized Water (mL)	Emulsion Containing Curcumin Nanocapsules (g)
Control	500	15	0
Cur-1	500	10	5
Cur-2	500	5	10
Cur-3	500	0	15

**Table 2:** Determination of water content, solubility and swelling degree of the control film and the film with curcumin added at different concentrations.

Sample	Water Content [%]	Solubility [%]	Degree of Swelling [%]
Control	17.06 ± 0.58 ^a^ *	24.04 ± 1.34 ^a^	30.93 ± 1.33 ^a^
Cur-1	15.25 ± 0.56 ^b^	13.67 ± 0.49 ^b^	18.68 ± 0.60 ^b^
Cur-2	9.82 ± 0.64 ^c^	12.71 ± 0.74 ^b^	11.22 ± 0.91 ^c^
Cur-3	11.71 ± 0.74 ^d^	13.19 ± 0.69 ^b^	12.57 ± 0.76 ^c^

* mean values marked by the same letters do not differ statistically at the confidence level of *p* = 0.05 (Fisher’s test).

**Table 3:** Mechanical properties of the films obtained at 25 °C and 25% humidity.

Sample	Thickness (mm)	TS (MPa)	EAB (%)
Control	0.090 ± 0.012 ^c^ *	29.63 ± 3.35 ^b^	34.50 ± 2.55 ^a^
Cur-1	0.111 ± 0.023 ^b^	12.38 ± 6.77 ^c^	29.49 ± 12.56 ^b^
Cur-2	0.129 ± 0.007 ^b^	29.56 ± 4.75 ^a^	29.07 ± 3.83 ^b^
Cur-3	0.178 ± 0.038 ^a^	11.33 ± 4.40 ^d^	40.93 ± 4.36 ^a^

* mean values marked by the same letters do not differ statistically at the confidence level of *p* = 0.05 (Fisher’s test). TS—tearing strength, EAB—percent elongation at break.

**Table 4:** Measurement of color and transparency of the control film and the film with curcumin added at different concentrations.

Sample	L*(D65)	a*(D65)	b*(D65)	O (-)
Control	96.71 ± 0.19 ^b *^	−0.29 ± 0.05 ^b^	3.71 ± 0.24 ^d^	1.39 ± 0.06 ^d^
Cur-1	97.16 ± 0.10 ^a^	−1.07 ± 0.06 ^c^	8.32 ± 0.58 ^c^	7.09 ± 0.45 ^a^
Cur-2	94.14 ± 0.13 ^d^	0.49 ± 0.04 ^a^	17.23 ± 0.21 ^a^	5.85 ± 0.18 ^b^
Cur-3	95.65 ± 0.54 ^c^	−1.21 ± 0.16 ^d^	11.02 ± 1.66 ^b^	3.12 ± 0.24 ^c^

* mean values marked by the same letters do not differ statistically at the confidence level of *p* = 0.05 (Fisher’s test)

**Table 5:** Wetting angles and surface free energy (SFE) for the control film and Cur-2 film.

Sample	Wetting Angles [°]		SFE [mJ/m^2^]		
	Water	DIM	Dispersion	Polar	Total
Control	79.98 ± 9.49	53.1 ± 5.40	32.53	5.08	37.61
Cur-2	67.94 ± 4.69	41.05 ± 5.15	39.08	8.55	47.63

**Table 6:** Particle/aggregate sizes (DLS) and zeta potential of the control film and the Cur-2 film.

**Sample**	**Zeta Potential [mV].**	**Particle Size [nm]**
Control	−41.3	8509
Cur-2	−37.8	5130

**Table 7:** Antimicrobial activity of the control and Cur-2 film (zone of inhibition diameter, mm).

Sample	*E. coli*	*A. fumigatus*	*P. expansum*
Control	0.0 ± 0.0	0.0 ± 0.0	0.0 ± 0.0
Cur-2	24.3 ± 1.2	21.3 ± 0.5	3.0 ± 1.1
Positive control	20.5 ± 1.5	22.3 ± 0.6	20.3 ± 0.6

## Data Availability

The data presented in this study are available upon request from the corresponding author.
